# Ambulance Dogs

**Published:** 1916-04-22

**Authors:** 


*To the Editor of* The Hospital.

### Ambulance Dogs.

*To the Editor of* The Hospital.

Sir,—With reference to your question on this subject at the foot of page 34, the Matrons' Section, of your issue for April 8, so far as my personal experience goes the British Army does not use ambulance dogs. I was at the Front during the whole of the mobile operations of the present campaign, and never saw such an animal. I think it is evident that Red Cross dogs would be valueless in the trench warfare which has gone on so long; and I would go a good deal further than this, for I doubt whether they are at any time worth their keep. I have seen the greatest (or at any rate the best advertised) breeder and exponent of ambulance dogs in this country give a public exhibition of their capacities; and I was impressed by nothing so much as the futility of the whole proceedings and the utter failure of the dogs to afford any real help. Your paragraph states that the Germans claim to have saved 4,000 lives by their use of dogs. I do not know whether the Germans do make this claim—doubtless your statement is made upon good authority—but if they do I can imagine no more excellent reason for disbelieving it. I fancy that astute breeders in this country sold great quantities of ambulance dogs to foreign Powers (before the war): but I am inclined to think that our own War Office did wisely not to adopt this policy, and that many of the incidents which have obtained publicity concerning Red Cross dogs are exaggerated or even apocryphal.—I am, Sir, yours faithfully,

Miles.

